# A molecular pore spans the double membrane of the coronavirus replication organelle

**DOI:** 10.1126/science.abd3629

**Published:** 2020-08-06

**Authors:** Georg Wolff, Ronald W. A. L. Limpens, Jessika C. Zevenhoven-Dobbe, Ulrike Laugks, Shawn Zheng, Anja W. M. de Jong, Roman I. Koning, David A. Agard, Kay Grünewald, Abraham J. Koster, Eric J. Snijder, Montserrat Bárcena

**Affiliations:** 1 Section Electron Microscopy, Department of Cell and Chemical Biology, Leiden University Medical Center, Leiden 2333 ZC, Netherlands.; 2 Molecular Virology Laboratory, Department of Medical Microbiology, Leiden University Medical Center, Leiden 2333 ZA, Netherlands.; 3 Department of Structural Cell Biology of Viruses, Centre for Structural Systems Biology, Heinrich Pette Institute, Leibnitz Institute of Experimental Virology, 22607 Hamburg, Germany.; 4 Howard Hughes Medical Institute, Department of Biochemistry and Biophysics, University of California San Francisco, San Francisco, CA 94143, USA.; 5 Department of Biochemistry and Biophysics, University of California San Francisco, San Francisco, CA 94143, USA.; 6 Department of Chemistry, MIN Faculty, Universität Hamburg, 20146 Hamburg, Germany.

## Abstract

Coronavirus genome replication is associated with virus-induced cytosolic double-membrane vesicles, which may provide a tailored microenvironment for viral RNA synthesis in the infected cell. However, it is unclear how newly synthesized genomes and messenger RNAs can travel from these sealed replication compartments to the cytosol to ensure their translation and the assembly of progeny virions. In this study, we used cellular cryo–electron microscopy to visualize a molecular pore complex that spans both membranes of the double-membrane vesicle and would allow export of RNA to the cytosol. A hexameric assembly of a large viral transmembrane protein was found to form the core of the crown-shaped complex. This coronavirus-specific structure likely plays a key role in coronavirus replication and thus constitutes a potential drug target.

## A gateway to the cytosol

Coronaviruses transform host cell membranes into peculiar double-membrane vesicles that have long been thought to accommodate viral genome replication. However, because these compartments appeared to be completely sealed, it has remained unknown how the newly made viral RNA could be exported to the cytosol for translation and packaging into new virions. Wolff *et al.* used cryo–electron microscopy to identify a molecular pore that spans the double membrane (see the Perspective by Unchwaniwala and Ahlquist). Six copies of a large coronavirus transmembrane protein formed the core of this structure, which may constitute a viral RNA export channel and provide a target for future antiviral interventions.

*Science*, this issue p. 1395; see also p. 1306

## Teaser

Viral RNA exported from the coronavirus replication organelle is likely to be mediated by a crown-shaped molecular pore.

Severe acute respiratory syndrome coronavirus 2 (SARS-CoV-2) is the third and most impactful example of a potentially lethal coronavirus infection in humans within the past 20 years (*1*–*3*). Coronaviruses are positive-stranded RNA (+RNA) viruses that replicate their unusually large genomes in the host cell’s cytoplasm. This process is supported by an elaborate virus-induced network of transformed endoplasmic reticulum (ER) membranes known as the viral replication organelle (RO) (*4*–*7*). Double-membrane vesicles (DMVs) are the RO’s most abundant component and the central hubs for viral RNA synthesis (*5*). The DMV’s interior accumulates double-stranded (ds) RNA, presumably intermediates of viral genome replication and subgenomic mRNA synthesis (*4*, *5*). DMVs may offer a favorable microenvironment for viral RNA synthesis and may shield viral RNA from innate immune sensors that are activated by dsRNA. However, coronaviral DMVs have been characterized as compartments that lack openings to the cytosol (*4*–*6*), despite the fact that newly-made viral mRNAs need to be exported for translation. Moreover, the coronavirus genome needs to be packaged by the cytosolic nucleocapsid (N) protein before being targeted to virus assembly sites on secretory pathway membranes (*8*).

**Fig. 1:**
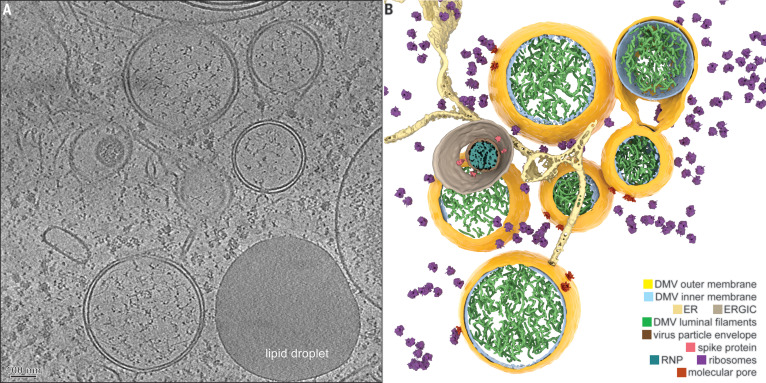
Coronavirus-induced DMVs revealed by cryo-ET. (**A**) Tomographic slice (7 nm thick) of a cryo-lamella milled through an MHV-infected cell at a middle stage of infection. (**B**) Three-dimensional (3D) model of the tomogram, with the segmented content annotated. See also movie S1. ERGIC, ER-to-Golgi intermediate compartment.

In this study, we used cryo–electron microscopy (cryo-EM) to analyze the structure of coronavirus-induced ROs in their native host cellular environment. The murine hepatitis coronavirus (MHV) is a well-studied model for the genus *Betacoronavirus*, which also includes severe acute respiratory syndrome coronavirus (SARS-CoV), Middle East respiratory syndrome coronavirus (MERS-CoV), and SARS-CoV-2. One advantage of MHV over these class 3 agents is the absence of serious biosafety constraints, thus making MHV suitable for in situ cryo-EM studies. We performed electron tomography (ET) on cryo-lamellae prepared by focused ion beam milling of cells in the middle stage of MHV infection. The tomograms revealed abundant perinuclear DMVs with an average diameter of 257 ± 63 nm (±SD), occasionally interconnected or connected to the ER as part of the reticulovesicular network described in previous work (Fig. 1, fig. S1, and movie S1) (*4*–*7*). In addition, macromolecular features that had not been discerned in conventional EM samples became apparent (figs. S2 to S4). The DMV lumen appeared to primarily contain filamentous structures that likely correspond to viral RNA (Fig. 1 and fig. S4). In part, this is expected to be present as dsRNA (*4*, *5*), as supported by the relatively long, straight stretches observed in some of these filaments, consistent with the persistence length of dsRNA (*9*) (fig. S4).

**Fig. 2:**
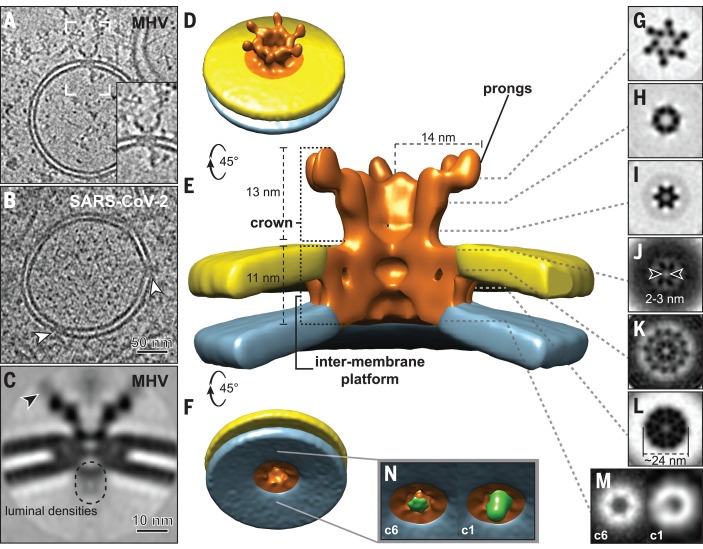
Architecture of the molecular pores embedded in DMV membranes. Tomographic slices (7 nm thick) revealed that pore complexes were present in both (**A**) MHV-induced DMVs and (**B**) prefixed SARS-CoV-2–induced DMVs (white arrowheads). The inset in (A) is a close-up view of the area delineated by white brackets. (**C** to **L**) Sixfold-symmetrized subtomogram average of the pore complexes in MHV-induced DMVs. (C) Central slice through the average, suggesting the presence of flexible or variable masses near the prongs (black arrowhead) and on the DMV luminal side. (D to F) Different views of the 3D surface-rendered model of the pore complex (copper colored) embedded in the outer (yellow) and inner (blue) DMV membranes. (G to L) 2D cross-section slices along the pore complex at different heights (see also movie S2). (**M** and **N**) An additional density at the bottom of the sixfold-symmetrized volume (c6, green) appeared as an off-center asymmetric density in the unsymmetrized average (c1).

Each DMV contained multiple copies of a molecular complex that spanned both membranes, connecting the DMV interior with the cytosol (Fig. 2A and supplementary text). Such complexes were also found in DMVs in prefixed SARS-CoV-2–infected cells (Fig. 2B and fig. S5). We surmise that this pore represents a generic coronaviral molecular complex that has a pivotal role in the viral replication cycle. Most likely, it allows the export of newly synthesized viral RNA from the DMVs to the cytosol. Functionally analogous viral complexes used for RNA export include those in the capsids of the *Reoviridae* (*10*) and, notably, the molecular pore in the neck of the invaginated replication spherules induced by flock house virus (*11*). None of these complexes, however, are integrated in a double-membrane organelle.

Subtomogram averaging of the double-membrane–spanning complexes in MHV-induced DMVs revealed an overall sixfold symmetry (Fig. 2, fig. S6, and movie S2). A cytosolic crown-like structure extended ~13 nm into the cytosol and was based on a ~24-nm-wide platform embedded in the DMV membranes. The two membranes did not fuse and maintained the typical DMV intermembrane spacing of ~4.5 nm (fig. S2). The complex formed a channel that followed its sixfold axis. On the DMV luminal side, the channel started with a ~6-nm-wide opening, narrowed toward the cytosol, and had two tight transition points (Fig. 2, J and L). The one at the level of the DMV outer membrane (Fig. 2J) was the most constricted, with an opening of ~2 to 3 nm, but would still allow the transition of RNA strands. Toward the cytosolic space, the complex opened into a crown-like structure, exposing six cytosolic “prongs.” With an achieved resolution of 3.1 nm, we roughly estimate that the complex has a total molecular mass of 3 MDa, of which the crown represents ~1.2 MDa (fig. S6).

**Fig. 3:**
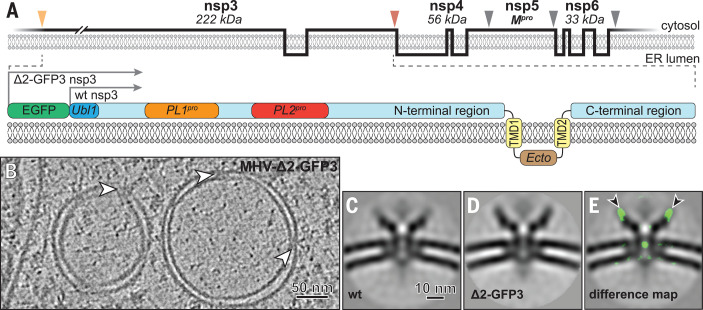
The coronavirus transmembrane protein nsp3 is a component of the pore complex. (**A**) (Top) Membrane topology of MHV transmembrane nsps, with protease cleavage sites indicated by orange (PL1^pro^), red (PL2^pro^), and gray (M^pro^) arrowheads. (Bottom) Detailed depiction of nsp3, showing some of its subdomains and the position of the additional EGFP moiety present in MHV-Δ2-GFP3. PL^pro^, papain-like protease; M^pro^, main protease. (**B**) Tomographic slice of DMVs induced by MHV-Δ2-GFP3, with embedded pore complexes (white arrowheads). (**C** and **D**) Comparison of the central slices of the sixfold-symmetrized subtomogram averages of the pore complexes in DMVs induced by (C) wild-type (wt) MHV and (D) MHV-Δ2-GFP3. (**E**) Density differences of 3 standard deviations between the mutant and wild-type structures, shown as a green overlay over the latter, revealed the presence of additional (EGFP) masses in the mutant complex (black arrowheads; see also movie S3).

We then considered the possible constituents of this complex. Coronaviruses express two large replicase polyproteins that are proteolytically cleaved into 16 nonstructural proteins (nsps) (*12*). Three of these nsps—nsp3 (222 kDa in MHV), nsp4 (56 kDa), and nsp6 (33 kDa)—are transmembrane proteins and thus are potential components of the pore. These nsps contain two, four, and six transmembrane domains (TMDs), respectively (*13*–*15*) (Fig. 3A), and engage in diverse homotypic and heterotypic interactions (*16*) thought to drive the formation of double-membrane ROs (*17*–*19*). On the basis of its size, the multidomain MHV nsp3 subunit is an attractive putative constituent of the pore. MHV nsp3 consists of a large cytosolic region of ~160 kDa, followed by two TMDs and a C-terminal cytosolic domain of ~41 kDa (*13*). Whereas the TMDs and C-terminal domain are highly conserved, the domain composition and size of the N-terminal part of nsp3 is quite variable among coronaviruses (*16*, *20*). Several nsp3 domains, including the conserved N-terminal ubiquitin-like domain 1 (Ubl1; 12.6 kDa) that binds both single-stranded RNA (*21*) and the N protein (*22*, *23*), may interact with viral RNA (*16*).

To investigate whether nsp3 is a component of the DMV molecular pore, we imaged cells infected with a well-characterized engineered MHV expressing nsp3 with an enhanced green fluorescent protein (EGFP) moiety fused to the Ubl1 domain [MHV-Δ2-GFP3 (*24*)] (fig. S7). This mutant lacks nsp2, which is dispensable for replication in cell culture (*25*). Subtomogram averaging of the pore complexes in these samples (Fig. 3B) revealed the presence of six additional densities on top of the prongs, each representing a mass compatible with that of EGFP (Fig. 3, C to E, and movie S3). These results identified nsp3 as a major constituent of the complex and provided insight into its orientation, with the Ubl1 domain residing in the prongs. Six copies of nsp3 can be envisioned to constitute most of the cytosolic crown-like structure (~1.2 MDa). Other viral and/or host proteins and lipids are probably also part of the ~1.8-MDa intermembrane platform, with nsp4 and nsp6 being prominent candidates. Notably, different studies suggest that nsp3-nsp4 interactions drive membrane pairing and determine DMV biogenesis and morphology (*17*–*19*, *26*).

The molecular pores frequently appeared to interact with other macromolecules on both the cytosolic and DMV luminal sides (fig. S8). In the subtomogram averages, these appeared as largely blurred out densities (Fig. 2C), which suggests that the interactions are dynamic. A small region on the luminal side, however, had a relatively higher density and was resolved in the unsymmetrized average as a closely associated and slightly off-center mass (Fig. 2, M and N, and fig. S6D). We speculate that this mass may be part of the viral replication machinery. The coronaviral replication-transcription complex (RTC) is thought to consist of a subset of relatively small (~10 to 110 kDa) nsps, with the RNA-dependent RNA polymerase (nsp12) at its core (*27*–*29*). However, some of these subunits may associate with the RTC only transiently, and the nsp stoichiometries of the complex are unknown. The luminal partners of the pore complex, prominent as masses varying in shape and size, appeared to interact with the putative RNA content of the DMVs (fig. S8).

The interaction partners of the cytosolic nsp3 prong ranged from chain-like masses to larger assemblies (fig. S8, black arrowheads). The subdomains of the long N-terminal nsp3 domain engage in a range of viral and virus-host interactions (*16*, *20*); consequently, the list of possible interactors is substantial. Among them, the viral N protein (55 kDa), which binds to the nsp3 Ubl1 domain (*22*, *23*), is a prominent candidate. The Ubl1-N interaction has been proposed to target viral RNA to replication sites at early stages of infection (*23*), but it may also modulate RNA exit and encapsidation on the cytosolic side of the pore complex. Notably, DMV-rich regions of the cytosol were crowded with protein assemblies that had a diameter of ~15 nm (fig. S9). These proteins strongly resembled the nucleocapsid structure in coronavirus particles, a helical ribonucleoprotein (RNP) complex that consists of the RNA genome and N protein oligomers (*30*) (fig. S9).

**Fig. 4:**
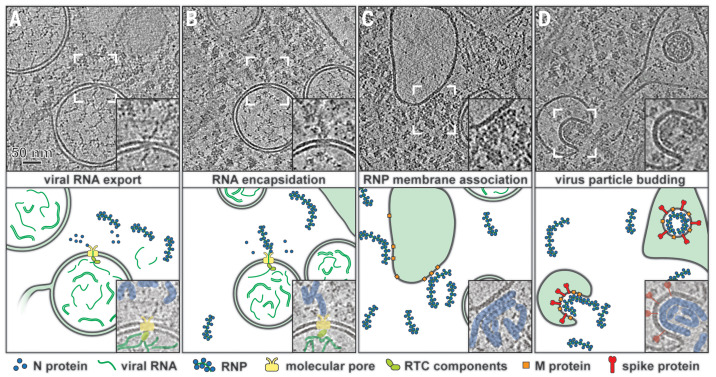
Model of the coronavirus genomic RNA transit from the DMV lumen to virus budding sites. Tomographic slices from MHV-infected cells (top) highlight the respective steps in the model (bottom). (**A**) The molecular pore exports viral RNA into the cytosol, (**B**) where it can be encapsidated by N protein. (**C**) Cytosolic RNP complexes can then travel to virus assembly sites for membrane association and (**D**) subsequent budding of virions. The insets in the top panels provide close-up views of the areas delineated by white brackets.

Our findings suggest a pathway for newly made viral genomic RNA from the DMV interior, via the channel of the pore, to the cytosolic sites of encapsidation. In our model, specific replicase subunits may associate with the pore complex to guide the newly synthesized RNA toward it (Fig. 4A). As proposed for other +RNA viral ROs (*11*), only +RNAs would need to be exported, whereas negative-stranded templates and/or dsRNA intermediates could remain inside the DMVs. On the cytosolic side, all exported viral mRNAs may associate with the N protein (Fig. 4B). Alternatively, the accumulating N protein could serve to select part of the newly made genomes for packaging. The remainder would then be used for translation, together with the much smaller, though much more abundant, subgenomic mRNAs (*31*). Genome-containing RNP complexes would travel to the membranes where the viral envelope proteins accumulate and engage in the assembly of progeny virions (Fig. 4C) (*32*). These bud into single-membrane compartments (Fig. 4D), typically derived from the ER-to-Golgi intermediate compartment (*8*), and travel along the secretory pathway to be released into extracellular space.

The double-membrane–spanning molecular pore revealed here may constitute the exit pathway for coronaviral RNA products from the DMV’s interior toward the cytosol, with the large and multifunctional nsp3 being its central component. Although the exact mode of function of this molecular pore remains to be elucidated, it seems to be a key structure in the viral replication cycle that is likely conserved among coronaviruses and thus may offer a coronavirus-specific drug target.

## Supplementary Materials

science.sciencemag.org/content/369/6509/1395/suppl/DC1 Materials and Methods Supplementary Text Figs. S1 to S9 References (*33*–*50*) MDAR Reproducibility Checklist Movies S1 to S3

## Appendix

View/request a protocol for this paper from *Bio-protocol*.
